# Malnutrition Prevalence Rates among Dutch Nursing Home Residents: What Has Changed over One Decade? A Comparison of the Years 2009, 2013 and 2018

**DOI:** 10.1007/s12603-021-1668-5

**Published:** 2024-01-04

**Authors:** Irma H.J. Everink, J.C.M. van Haastregt, M. Manders, M.A.E. de van der Schueren, J.M.G.A. Schols

**Affiliations:** 1 Department of Health Services Research and Care and Public Health Research Institute (CAPHRI), Maastricht University, Maastricht, the Netherlands; 2 Department of Nutrition, Dietetics and Lifestyle, School of Allied Health, HAN University of Applied Sciences, Nijmegen, the Netherlands; 3 Division of Human Nutrition and Health, Wageningen University and Research, Wageningen, the Netherlands; 4 Department of Family Medicine and Care and Public Health Research Institute (CAPHRI), Maastricht University, Maastricht, the Netherlands

**Keywords:** Malnutrition, long-term care, nutritional status, aged

## Abstract

**Objectives:**

To assess changes in prevalence of malnutrition and its associated factors among people living in Dutch nursing homes in 2009, 2013 and 2018.

**Design:**

Secondary data analysis of the International Prevalence Measurement of Care Quality (LPZ) study.

**Setting:**

Dutch nursing homes.

**Participants:**

Residents living at a psychogeriatric or somatic ward in Dutch nursing homes in 2009, 2013 or 2018.

**Measurements:**

weight and height, unintentional weight loss over the last month and last six months, age, sex, length of stay up to the measurement day, care dependency, and the presence of various diseases (dementia, diabetes mellitus, stroke, diseases of the respiratory system, respiratory diseases and pressure ulcers).

**Results:**

In total, 14,317 residents were included in this study with a mean age of 82.2, 70.9 female and 66.8% was living on a psychogeriatric ward. Results of this study show relative stability in background characteristics of the nursing home population over the last decade. In the total sample, 16.7% was malnourished and these percentages were 16.6% in 2009, 17.5% in 2013 and 16.3% in 2018. Multiple binary logistic regression analyses revealed having a pressure ulcer, female sex and living on a psychogeriatric department to be positively associated and having diabetes mellitus to be negatively associated with malnutrition throughout the years. These associations were strong and similar across years.

**Conclusion:**

Even though much attention has been paid to prevent malnutrition in Dutch nursing homes over the last decades, results show a relatively stable malnutrition prevalence rate of around 16%. This leads to the question if nursing staff is able to sufficiently recognize residents with (a risk of) malnutrition, and if they are aware of interventions they could perform to decrease this rate.

## Background

**M**alnutrition is a highly prevalent problem among nursing home residents worldwide, as shown by a systematic review indicating an average prevalence rate of 21.4% (1). Malnutrition is defined as “a state resulting from lack of intake or uptake of nutrition that leads to altered body composition (decreased fat free mass) and body cell mass leading to diminished physical and mental function and impaired clinical outcome from disease” (2). As the definition already suggests, malnutrition can have serious consequences, in particular for older adults. It leads to a weaker immune system and impaired wound healing (3, 4), a higher fall risk and impaired functionality (5) (6). Malnutrition also decreases quality of life, leads to higher health care costs and increases mortality risk (7, 8).

People living in long-term care facilities, such as nursing homes, have a higher risk to develop malnutrition due to factors such as cognitive problems, decreased motor skills, dysphagia, problems with vision and/or poor appetite (9, 10). As a result, ensuring adequate food intake in this population is challenging. Various interventions seem beneficial to reduce malnutrition in nursing homes, such as performing nutritional screening, educating staff, providing oral nutritional supplements, supervision during mealtime, having the resident choose the food of their preference and attention to mealtime ambiance (8, 11). However, these interventions require time and resources and changes in daily routines (10).

As the risk of malnutrition increases with age (12), the challenge of malnutrition in long-term care facilities might further increase with increasing life expectancy. It is assumed that residents in long-term care facilities are older nowadays and more care dependent compared to a decade ago. This expectation is based on the ‘ageing-in-place policy’ that many Western countries embrace, which requires that older adults stay at home as long as possible instead of moving to a long-term care facility (13). Before 2015, older adults living in the Netherlands with mild impairments could move to a residential home where they received assistance with tasks such as bathing or dressing but were rather independent in performing other activities. Older adults with more severe disabilities and a higher care dependency were living in nursing homes, where they received continuous (24 hour) assistance in activities of daily living but also medical care. Since 2015, most residential care facilities have been closed gradually. Nowadays, only nursing homes are open for people with severe physical or cognitive disabilities and in need of (medical) care (13). Older adults with less severe impairments now stay at home assisted by community care services and/or informal care. The stricter admission policy is expected to result in changes in patients' and disease characteristics of the nursing home population (older, more impaired) which could, in turn, result in changes in the prevalence of malnutrition and its associated factors.

It is known for many years that malnutrition is a problem among older adults residing in long-term care facility, which led to the increased attention towards prevention and treatment of malnutrition among policy makers and scientists. This resulted in the development, implementation and evaluation of various (previously described) interventions such as nutritional screening, providing nutritional supplements, finger foods, attention to mealtime ambiance and educating staff (14, 15). However, it is unknown if this increased attention towards prevention and treatment of malnutrition resulted in lower malnutrition prevalence rates.

Therefore, the aim of this study is to assess the prevalence of malnutrition and its associated factors among people living in Dutch nursing homes in 2009, 2013 and 2018. These three years are supposed to present the last decade. Providing insight into potential changes throughout the years could result in adapted recommendations for malnutrition prevention and treatment in nursing homes

## Methods

### Study design

This study is a secondary data analysis using data from the International Prevalence Measurement of Care Quality (LPZ) study (16). This study uses a cross-sectional design to perform a yearly prevalence measurement of six different care problems among patients and residents in hospitals, nursing homes and home care. The care problems under consideration are malnutrition, pressure injuries, continence, falls, restraints and pain. Besides measuring the prevalence of these care problems, the measures taken to prevent and treat these care problems are measured. Furthermore, the LPZ measures a range of demographic variables of participants such as age, gender, length of stay and care dependency. When organizations participate in the LPZ measurement, they receive information in digital dashboards about their own prevalence rates on the different care problems, as well as information on which preventive measures and treatment options they have taken. This information is provided at organizational level, location level and ward level, enabling internal benchmarking. Next to information about performance of their own organization, location and wards, they also receive external benchmarking information. This includes the aggregated results of comparable organizations participating in LPZ. The LPZ project team receives the anonymized results to perform scientific research.

Participation in the LPZ study is voluntarily and decided on organizational level. If an organization decides to participate, they have to include all locations and departments of their organization. Since 2016, organizations can decide themselves which care problems they want to measure. All patients/residents have to give consent for participation in the LPZ study.

### Participants

Participants in this study include residents living in a nursing home in the Netherlands. We included participants from the years 2009, 2013 and 2018, to be able to study potential changes in residents' characteristics, disease severity, and malnutrition prevalence rates. Nursing homes are long-term care facilities for older adults with severe physical or cognitive disabilities (such as dementia) with a need for continuous (medical) care or support. To ensure comparability of data, only the largest ward types in Dutch nursing homes, wards for psychogeriatric care and somatic care respectively, were included in this analysis.

To be included in the study, participants had to give consent to participate in the LPZ study. In the cases of people with dementia, their legal representative had to give consent to participate. Also, participants had to be living at a psychogeriatric or somatic ward in a nursing home in the Netherlands in 2009, 2013 or 2018, and had to be 18 years or older. Lastly, to be included in the study, participants needed to have all data measured to calculate the malnutrition rate. If there was data missing (e.g. on weight or length), the participant was excluded from the analysis.

### Ethical approval

The LPZ study is approved by the Medical Ethics Committee of University Hospital Maastricht (MEC 01–014). Each participant was informed about the content of the study and had to give oral informed consent. All data was collected anonymously and treated as strictly confidential.

### Variables

#### Malnutrition variables

To calculate whether or not a resident suffers from malnutrition, data on current weight (kg), current height (cm), unintential weight loss in the last month (yes/no), unintential weight loss in the last six months (yes/no), the percentage of unintential weight loss in the last month and the percentage of unintential weight loss over the last six months were measured. This data was used to calculate the actual malnutrition rate, according to the international ESPEN definition (2) (box 1). In 2009 and 2013, LPZ participants completed the full questionnaire including background characteristics and questions of all care problem modules. In 2018, background characteristics were obliged for all participants but organizations could choose to participate in only the care problem modules (e.g. malnutrition or pressure ulcers) of their interest. As a consequence, not all organizations participating in the 2018 measurement have data on malnutrition.

##### Box 1 Criteria to assess malnutrition used in this study, based on ESPEN definition(2)

A person suffers from malnutrition if:

• BMI <18 kg/m^2^ for people <65 years of age, and, BMI <20 kg/m^2^ for people ≥65 years of age, or
• >5% weight loss in the last month, or
• >10% weight loss in the last 6 months

#### Background variables

To assess which variables were associated with malnutrition, various background variables were included in the analyses. The background characteristics were selected based on known risk factors for malnutrition risk from the in literature, on expert opinion and on availability in the LPZ dataset.

The background characteristics included are age (years), sex, length of stay in the long-term care facility up to the measurement day (days) and care dependency. Care dependency was measured using the Care Dependency Scale, a 15-item instrument for need assessment and care planning of institutionalized patients (17). This scale assesses a residents' care dependency on several domains, such as eating and drinking, continence, body posture, mobility, communication, etc. Each domain (item) is assessed with a score from 1 to 5, where 1 represents ‘completely dependent’ and 5 represents ‘(almost) independent’. The CDS sum score ranges from 15 (completely dependent) to 75 (completely independent). Next to the CDS sum score, the single CDS items ‘eating and drinking’, ‘communication’ and ‘mobility’ were included in this analysis as it is expected that these single items could have a larger influence on malnutrition rate compared to the other items of the scale. Furthermore, the presence or absence of five ICD-10 diseases were measured: having dementia (yes/no), having diabetes mellitus (yes/no), having a stroke (yes/no), having diseases of the nervous system (yes/no) and having respiratory diseases (yes/no). Finally, suffering from a pressure ulcer (yes/no) and the type of ward where the resident was living at (psychogeriatric nursing home ward or somatic nursing home ward) were included as variables in this analysis.

### Data collection

Primary data collection was performed by participating organizations themselves. All organizations appointed a coordinator who was trained how to perform data collection by the LPZ study team trainer. This coordinator was responsible for coaching the raters of his own organization how to perform data collection. All participating organizations received all questionnaires, instruction materials and a manual from the LPZ study team. The participating organizations could choose to perform the prevalence measurement in April or November of the respective year. For reliability purposes, measuring the residents was done by pairs of trained nurses. Of these pairs, one rater was working on the respective ward and one rater was working on a different ward. The two raters had to reach agreement on their answers to all questions. In the cases no agreement was reached, the answer of the independent rater (from the other ward) prevailed. All data was entered into the digital entry system developed and hosted by Flycatcher Internet Research B.V. in Maastricht, the Netherlands.

### Data analysis

Data was analyzed using SPSS for Windows version 25. Describing the sample and comparing the groups with and without malnutrition on background characteristics was done using descriptive statistics (mean, median and percentages), mean differences and odds ratios. Mean differences and odds ratios included 95% confidence intervals. Background characteristics of residents in the years 2009, 2013 and 2018 datasets were compared using one-way Anova analyses (continuous data) or Chi-square tests (binary data).

Besides describing the full sample, separate analyses were performed per year (2009, 2013 and 2018). For each year, the group with and group without malnutrition was described and compared on background characteristics using mean differences and odds ratios including 95% confidence intervals. Furthermore, all variables which were associated with malnutrition in the full sample (proven by the fact that the confidence interval did not include zero in the case of mean differences, or that the confidence interval did not include one (1) in the case of odds ratios) were entered in a multiple binary logistic regression analysis per year. The variance inflation factor (VIF) was calculated to determine collinearity between variables. A VIF ≤9 was assessed as being acceptable. Finally, all participants with one or more missing variables were excluded from the multivariate analysis.

## Results

Table 1 shows the characteristics of the total sample (n=14,317). The three samples differed in size, caused by variations in ward types throughout the years. The general age of the total sample was 82.2 years and 70.9% was female. The median length of stay in the nursing home at the day of measurement was 683 days. Out of the medical diagnoses included in this study, dementia was the most prevalent (65.9%), also shown by the proportion of residents living in a psychogeriatric department (66.8%). The prevalence rate of malnutrition was 16.7% in the total sample. This percentage was 16.6% in 2009, 17.5% in 2013 and 16.3% in 2018. As shown in table 1, there were statistical differences in sample characteristics in the nursing home population between the three years. The mean age slightly increased from 82.0 years in 2009 to 81.9 years in 2013 and 83.5 years in 2018 (p<0.001) and the mean care dependency slightly decreased from 36.9 in 2009 to 38.2 in 2013 and 42.5 in 2018 (a higher score represents less dependency). For all other variables, there were no clear increasing or decreasing trends: often scores increased in 2013 compared to 2009, and decreased again in 2018 compared to 2013.

**Table 1:** Background characteristics of total sample and samples per year (2009, 2013 and 2018)

	**Total sample (n=14,317)**	**Sample 2009 (n=8,195)**	**Sample 2013 (n=3,194)**	**Sample 2018 (n=2,928)**	**p-value**
Type of department, n (%)					<0.001
Psychogeriatrics	9,558 (66.8%)	5,575 (68.0%)	2,106 (65.9%)	1,877 (64.1%)	
Somatic	4,759 (33.2%)	2,620 (32.0%)	1,088 (34.1%)	453 (15.5%)	
Malnutrition, n (%)	2,394 (16.7%)	1,357 (16.6%)	559 (17.5%)	478 (16.3%)	0.390
Age (years)					<0.001
Mean (SD)	82.2 (9.4)	82.0 (9.2)	81.9 (9.6)	83.2 (9.5)	
Median (IQR)	84.0 (78.0–84.0)	83.0 (78.0 – 88.0)	84.0 (77.0 – 88.0)	85.0 (79.0 – 90.0)	
Female, n (%)	10,151 (70.9%)	5,879 (71.7%)	2,226 (69.7%)	2,046 (69.9%)	0.038
BMI (kg/m^2^)					<0.001
Mean (SD)	24.9 (5.4)	24.9 (5.1)	24.6 (5.0)	25.1 (6.3)	
Median (IQR)	24.3 (21.4 – 27.6)	24.4 (21.5 – 27.8)	24.0 (21.1 – 27.3)	24.2 (21.5 – 27.5)	
Length of admission (days)					0.001
Mean (SD)	988 (1,028)	965 (1,007)	993 (1,039)	1,050 (1,073)	
Median (IQR)	683 (271 – 1372)	667 (272 – 1343)	698 (278 – 1367)	715 (263 – 1450)	
CDS Sum score (range 15–75)					<0.001
Mean (SD)	38.4 (17.2)	36.9 (17.1)	38.2 (17.7)	42.5 (16.2)	
Median (IQR)	36.0 (23.0 – 52.0)	34.0 (22.0 – 50.0)	36.0 (22.0 – 52.0)	43.0 (30.0 – 55.0)	
CDS item ‘eating and drinking’ (range 1–5)					<0.001
Mean (SD)	2.7 (1.4)	2.6 (1.4)	2.6 (1.5)	3.1 (1.3)	
Median (IQR)	2.0 (1.0 – 4.0)	2.0 (1.0 – 4.0)	2.0 (1.0 – 4.0)	3.0 (2.0 – 4.0)	
CDS item ‘communication’ (range 1–5)					<0.001
Mean (SD)	3.3 (1.5)	3.3 (1.5)	3.2 (1.5)	3.6 (1.4)	
Median (IQR)	4.0 (2.0 – 5.0)	3.0 (2.0 – 5.0)	3.0 (2.0 – 5.0)	4.0 (3.0 – 5.0)	
CDS item ‘mobility’ (range 1–5)					<0.001
Mean (SD)	2.8 (1.6)	2.7 (1.6)	2.8 (1.6)	3.0 (1.5)	
Median (IQR)	3.0 (1.0 – 4.0)	2.0 (1.0 – 4.0)	3.0 (1.0 – 4.0)	3.0 (2.0 – 4.0)	
Diagnoses, n (%)					
Dementia	9,437 (65.9%)	5,521 (67.4%)	2,087 (65.3%)	1,829 (62.5%)	<0.001
Diabetes mellitus	2,714 (19.0%)	1,485 (18.1%)	639 (20.0%)	590 (20.2%)	0.013
Diseases of the nervous system	1,697 (11.9%)	888 (10,8%)	446 (14.0%)	363 (12.4%)	0.000
Stroke	3,243 (22.7%)	1,928 (23.5%)	771 (24.1%)	544 (18.6%)	<0.001
Respiratory diseases	1,884 (13.2%)	950 (11.6%)	481 (15.1%)	453 (15.5%)	<0.001
Pressure ulcer, n (%)	1,523 (10.8%)	1,074 (13.1%)	181 (5.7%)	268 (9.9%)	

Table 2 shows the characteristics of the total group of subjects with and without malnutrition. It appears that subjects with malnutrition had a higher age, were more often female, were overall more care dependent (lower score on CDS), which is also shown by higher care dependency on the single items ‘eating and drinking’, ‘communication’ and ‘mobility’, and were more often affected by dementia and pressure ulcers. Furthermore, subjects with malnutrition were less often affected by diabetes mellitus and stroke. Lastly, being admitted to a psychogeriatric department was positively associated with malnutrition whilst being admitted to a somatic department, was negatively associated with malnutrition. Even though borderline significant, suffering from diseases of the nervous system and of respiratory diseases were not associated with malnutrition, neither was length of stay.

**Table 2:** Comparison of groups with and without malnutrition on background characteristics

	**Without malnutrition (n=11,923)**	**With malnutrition (n=2,394)**	**Mean difference (95% CI)**	**Odds ratio (95% CI)**
Age, years (SD)	81.9 (9.5)	84.0 (8.6)	2.1 (1.7 to 2.4)*	n.a.
Female, n (%)	8,237 (69.1%)	1,914 (79.9%)	n.a.	1.8 (1.6 – 2.0)*
BMI, kg/m^2^ (SD)	26.1 (4.9)	18.7 (2.5)	−7.4 (−7.6 to −7.2)*	n.a.
Length of admission, days (median)	988.9 (1028.2)	985.3 (1027.5)	P=0.383 (U-test)	n.a.
CDS Sum score (mean)	39.5 (17.0)	32.7 (16.7)	−6.8 (−7.6 to −6.1)*	n.a.
CDS item ‘eating and drinking’ (mean)	2.8 (1.4)	2.3 (1.4)	−0.5 (−0.6 to −0.5)*	n.a.
CDS item ‘communication’ (mean)	3.4 (1.5)	2.9 (1.5)	−0.5 (−0.6 to −0.5)*	n.a.
CDS item ‘mobility’ (mean)	2.8 (1.6)	2.4 (1.6)	−0.4 (−0.5 to −0.4)*	n.a.
Dementia, n (%)	7,664 (64.3%)	1,773 (74.1%)	n.a.	1.6 (1.4 – 1.8)*
Diabetes mellitus, n (%)	2,405 (20.2%)	309 (12.9%)	n.a.	0.6 (0.5 – 0.7)*
Diseases of the nervous system	1,437 (12.1%)	260 (10.9%)	n.a.	0.9 (0.8 – 1.0)
Stroke	2,796 (23.5%)	447 (18.7%)	n.a.	0.8 (0.7 – 0.8)*
Respiratory diseases	1,591 (13.3%)	293 (12.2%)	n.a.	0.9 (0.8 – 1.0)
Pressure ulcer, n (%)	1,153 (9.9%)	370 (15.8%)	n.a.	1.7 (1.5 – 2.0)*
Type of department, n (%)				
Psychogeriatric	7,754 (65.0%)	1,804 (74.4%)	n.a.	1.6 (1.4 – 1.8)*
Somatic	4,169 (35.0%)	590 (24.6%)	n.a.	0.6 (0.6 – 0.7)*


*Statistically significant difference

In appendix 1, results of the comparison of subjects with and without malnutrition can be found per year (2009, 2013 and 2018), as well as the multiple binary logistic regression analysis for factors associated with malnutrition of subjects per year (2009, 2013 and 2018).

When looking at the results of the 2009 measurement, It appears that malnutrition was positively associated with a higher age, being female, lower BMI, higher care dependency (both total sum score as the single items ‘eating and drinking’, ‘communication’ and ‘mobility‘), dementia, having a pressure ulcer and living on a psychogeriatric department. Malnutrition was negatively associated with having diabetes mellitus, and being admitted to a somatic department. Malnutrition was not associated with length of stay, diseases of the nervous system, stroke or respiratory diseases.

When looking at results from the multiple binary logistic regression analysis for factors associated with malnutrition of subjects participating in the 2009 measurement, it appears that pressure ulcers (OR 1.581; 95% CI 1.344 – 1.860), female sex (OR 1.498; 95% CI 1.287–1.744) and living on a psychogeriatric department (OR 1.281; 95% CI 1.009 – 1.628) were most strongly associated with malnutrition. A strong negative association was found for having diabetes mellitus (OR 0.570; 95% CI 0.475 – 0.683) and having had a stroke (OR 0.787; 95% CI 0.670 – 0.924).

Results from the 2013 measurement show positive associations between malnutrition and higher age, female sex, higher care dependency, dementia, pressure ulcers and being admitted to a psychogeriatric department. Negative associations are shown for malnutrition and BMI, diabetes mellitus and being admitted to a somatic department. The multiple regression analysis shows the strongest associations with malnutrition for female sex (OR 1.720; 95% CI 1.361 – 2.175), having a pressure ulcer (OR 1.632; 95% CI 1.131 – 2.356) and being admitted to a psychogeriatric department (OR 1.602; 95% CI 1.104 – 2.326). A strong negative association was found for diabetes mellitus (OR 0.579; 95% CI 0.443 – 0.755).

The 2018 measurement shows that age, female gender, lower BMI, higher care dependency, having a pressure ulcer and being in a psychogeriatric department are positively associated with malnutrition. Negative associations were found between malnutrition and having diabetes mellitus and living on a somatic department. The multiple binary logistic regression analysis shows the strongest associations with malnutrition for having a pressure ulcer (OR 1.549; 95% CI 1.121 – 2.141), female sex (OR 1.454; 95% CI 1.131 – 1.869) and living on a psychogeriatric department (OR 1.424; 95% CI 0.947 – 2.141).

## Discussion

The aim of this study was to assess the prevalence of malnutrition and factors associated with malnutrition among people living in Dutch nursing homes in 2009, 2013 and 2018. The prevalence rate of malnutrition, calculated using the ESPEN definition, was 16.7% in the total sample over the three years, and 16.6%, 17.5% and 16.3% for 2009, 2013 and 2018, respectively. Even though the sample characteristics of participants in the 2009, 2013 and 2018 measurement showed significant differences, only mean age showed a clear increasing trend and care dependency showed a clear decreasing trend. For all other variables (type of department, sex, BMI, length of admission, % dementia, % diabetes mellitus, % diseases of the nervous system, % stroke, % respiratory diseases and % pressure ulcers) there was no clear increasing nor decreasing trend. Often, scores increased in 2013 compared to 2009, and decreased again in 2018 compared to 2013.

As described in the introduction, due to the ‘ageing in place‘-policy, we expected to see increasing age, increasing care dependency and increasing malnutrition prevalence rates in the nursing home population over the years. Besides a significant increase in mean age, our results do not confirm this hypothesis and even contradict this in the case of care dependency, which appeared to decrease. Two possible explanations could be given for this finding. The first one is that the instrument used to measure care dependency, the Care Dependency Scale, measures items such as eating and drinking, continence and mobility, which might not be of equal relevance for somatic and psychogeriatric patients. For people living on somatic wards, it is likely that these items cause higher care dependency. However, it is also likely that on psychogeriatric wards, people are still rather independent on the aforementioned items, and higher care dependency among this group is caused by different issues such as agitation or restlessness. These behavioral problems might pose a larger burden on caregivers but are not captured by the CDS. Another explanation could be that in 2015, many residential care facilities closed and various residents were transferred from the residential care facility to a nursing home ward, even though they did not have the required severe (cognitive) impairments or high care dependency. This group could still be residing in these nursing homes in 2018 and causing the lower care dependency rates. This group could also explain the relatively large and stable length of stay.

Malnutrition prevalence rates did not significantly differ between the three years. This is surprising as there has been increasing attention towards malnutrition care in nursing homes in the last decade, both nationally and internationally (2, 15). To the best of our knowledge, there are no other studies describing malnutrition prevalence rates throughout the years among nursing home residents. Two studies performed in Belgian nursing homes where prevalence rates of malnutrition were measured in 2008 and 2013 showed similar relatively stable malnutrition prevalence rates of 15.9% in 2008 (18) and 14% in 2013 (12). These studies did use a different method to assess malnutrition (BMI<20 in 2008 and MNA-SF score in 2013), and in the 2008 measurement, no distinction was made between community-dwelling older adults and nursing home residents (18). However, this Belgian study also concludes that, despite all efforts from the government and nutrition-focused organizations, malnutrition remains a big problem in nursing homes (12).

A lot of research has been done towards the effects of interventions on malnutrition rates, such as consulting a dietician, providing dietary supplements, giving residents a larger role in their food choice, improving mealtime ambiance, staff training programs and structural screening (8, 11), and they all seem promising. As interventions seem to help but malnutrition rates do not decrease, the question arises if interventions are actually applied, and if nursing home staff is able to recognize residents suffering from (a risk of) malnutrition. As staff that used to work in residential care facilities now works in nursing homes, where a more care dependent population resides, it is important to see if they are sufficiently skilled to recognize malnutrition and know what preventive measures or interventions they need to take. A study of Vanderwoude et al. (2019) showed that nursing home staff still has difficulties recognizing (signs of) malnutrition: only 49% of all malnourished patients in the study population were recognized as malnourished (12). These numbers address the importance of (ongoing) governmental initiatives focusing on malnutrition in nursing homes, such as the Dutch Quality Framework for Nursing Homes, in which attention for nutrition is one of the main themes (19).

A last explanation for lack of a decrease in malnutrition rates could be that before 2009, malnutrition prevalence rates in nursing homes were higher compared to prevalence rates measured now and that we are dealing with a floor effect. A study of Meijers et al. (20), using the same LPZ database, showed malnutrition rates in nursing homes between the years 2004–2007 of around 25% (20). Comparing these rates with the malnutrition rate found in our study (16.7%) shows that a decrease in malnutrition rate was already achieved before 2009. Therefore, we could also ask ourselves the question if it is realistic to expect further improvement in this frail population.

Multiple binary logistic regression analyses revealed that factors showing the strongest positive associations with malnutrition were similar across years and were having a pressure ulcer, female sex and living on a psychogeriatric department. Having diabetes mellitus showed a strong negative association with malnutrition throughout the years. Our results on factors associated with malnutrition were partially confirmed by a systematic review from 2015 on factors associated with an increased risk of malnutrition (8). This study confirms an association between pressure ulcers and malnutrition but does not report female sex or living on a psychogeriatric ward as risk factors. However, dementia or cognitive impairment, diseases which are highly frequent on a psychogeriatric ward, are mentioned as important risk factors in this review. It is surprising that our study did not show a positive significant association between having a stroke or respiratory diseases with malnutrition, and that having a stroke was even negatively associated with malnutrition in 2009. This, while stroke and respiratory diseases are frequently mentioned as important risk factors for malnutrition. A possible explanation for this finding could be that during LPZ data collection, only the diagnosis was assessed which was most relevant for nursing home admission, instead of all (co)morbidities residents had, of which a a stroke or having a respiratory disease could be one.

### Strengths and limitations

Many studies on malnutrition prevalence rates in acute care, long-term care and community settings are performed. However, to the best of our knowledge, there are no studies that measure malnutrition throughout the years using the same methodology and definition of malnutrition. Therefore, this study provides valuable insights into (lack of) changes in malnutrition rates and its associated factors throughout the years.

A limitation of this study is that, by performing a secondary data analysis, only variables that were taken into account in the primary data collection can be included in our model. This means that we could have missed factors associated with malnutrition but that were not measured, such as depression, chewing problems or dysphagia. The latter two are now included in the LPZ methodology but not in 2009 and 2013 measurements and could therefore not be taken into account. Another limitation is that the participation rate over the years declined and not all organizations chose to include the malnutrition module during the 2018 measurement. There is a possibility that only due to higher malnutrition prevalence rates in preceding years, only organizations that already had more focus on malnutrition care chose to use the malnutrition module. However as we still have relatively large numbers of participants, we believe this did not influence our results to a great extent. Lastly, the LPZ study has a cross-sectional design, making it impossible to establish causal relationships.

## Conclusion

Even though much attention has been paid to prevent malnutrition in Dutch nursing homes over the last two decades, results of this study show relatively high and stable malnutrition prevalence rates around 16%. This leads to the question if nursing staff is able to sufficiently recognize residents with (a risk of) malnutrition, and if they are aware of interventions they could perform to decrease this rate, such as consulting a dietician, and if they are performing the interventions in the correct way. From our findings it also becomes clear that factors associated with malnutrition remained stable over the years, and include female gender, having a pressure ulcer and living on a psychogeriatric department. Extra attention should be paid on prevention of malnutrition among residents displaying this profile.

## Electronic supplementary material

Appendix 1: Malnutrition analyses in 2009, 2013 and 2018
