# Supplementary material for: Malnutrition Prevalence Rates among Dutch Nursing Home Residents: What Has Changed over One Decade? A Comparison of the Years 2009, 2013 and 2018
Source: J Nutr Health Aging. 2024 Jan 4;25(8):999–1005. doi: 10.1007/s12603-021-1668-5 (PMC12929994; doi:10.1007/s12603-021-1668-5)
Supplement: Supplementary file 1 — Appendix 1: Malnutrition analyses in 2009, 2013 and 2018 [file mmc1.docx]

**Appendix 1: Malnutrition analyses in 2009, 2013 and 2018**

**Table 1** Comparison of subjects with and without malnutrition admitted in 2009

|  | **Without malnutrition**  **(n=6,838)** | **With malnutrition**  **(n=1,357)** | **Mean difference (95% CI)** | **Odds ratio**  **(95% CI)** |
| --- | --- | --- | --- | --- |
| Age, years (SD) | 81.7 (9.3) | 83.4 (8.5) | 2.1 (1.7 to 2.7)* | n.a. |
| Female, n (%) | 4,786 (70.0%) | 1,093 (80.5%) | n.a. | 1.8 (1.5 to 2.1)* |
| BMI, kg/m^2^ (SD) | 26.2 (4.6) | 18.8 (2.6) | -7.4 (-7.6 to -7.2)* | n.a. |
| Length of admission, days (median) | 666 (1019.7) | 670 (943.5) | p= 0.964 (U-test) | n.a. |
| CDS Sum score | 38.2 (17.0) | 30.5 (16.1) | -7.7 (-8.6 to -6.7)* | n.a. |
| CDS item ‘eating and drinking’ | 2.7 (1.4) | 2.1 (1.3) | -0.6 (-0.7 to -0.5)* | n.a. |
| CDS item ‘communication’ | 2.1 (1.3) | 3.4 (1.5) | -0.6 (-0.7 to -0.5)* | n.a. |
| CDS item ‘mobility’ | 2.8 (1.6) | 2.3 (1.6) | -0.5 (-0.6 to -0.4)* | n.a. |
| Dementia, n (%) | 4,473 (65.4%) | 1,048 (77.2%) | n.a. | 1.8 (1.6 to 2.1)* |
| Diabetes mellitus, n (%) | 1,328 (19.4%) | 157 (11.6%) | n.a. | 0.5 (0.5 to 0.6)* |
| Diseases of the nervous system | 757 (11.1%) | 131 (9.7%) | n.a. | 0.9 (0.7 to 1.0) |
| Stroke | 1,670 (24.4%) | 258 (19.0%) | n.a. | 0.7 (0.6 to 0.8) |
| Respiratory diseases | 817 (11.9%) | 133 (9.8%) | n.a. | 0.8 (0.7 to 1.0) |
| Pressure ulcer, n (%) | 812 (11.9%) | 262 (19.4%) | n.a. | 1.8 (1.5 to 2.1)* |
| Type of department, n (%) |  |  |  |  |
| Psychogeriatric | 4,525 (66.2%) | 1,050 (77.4%) | n.a. | 1.7 (1.5 to 2.0)* |
| Somatic | 2,313 (33.8%) | 307 (22.6%) | n.a. | 0.6 (0.5 to 0.7)* |

*Statistically significant difference

**Table 2.** Multiple binary logistic regression analysis for factors associated with malnutrition of subjects admitted to nursing homes in 2009

| **Independent variables** | **Beta** | **Standard error** | **Wald statistics** | **OR (95% CI)** | **p-value** | **VIF** |
| --- | --- | --- | --- | --- | --- | --- |
| Age, years | 0.019 | 0.004 | 23.649 | 1.019 (1.011-1.026) | 0.000 | 1.132 |
| Female | 0.404 | 0.077 | 27.217 | 1.498 (1.287-1.744) | 0.000 | 1.074 |
| CDS item ‘eating and drinking’ | -0.207 | 0.030 | 46.322 | 0.813 (0.766-0.863) | 0.000 | 1.756 |
| CDS item ‘communication’ | -0.092 | 0.025 | 13.346 | 0.912 (0.868 – 0.958) | 0.000 | 1.625 |
| CDS item ‘mobility’ | -0.046 | 0.024 | 3.693 | 0.955 (0.910 – 1.001) | 0.055 | 1.588 |
| Dementia | 0.018 | 0.122 | 0.022 | 1.018 (0.801 – 1.294) | 0.882 | 3.105 |
| Diabetes mellitus | -0.563 | 0.092 | 37.103 | 0.570 (0.475 – 0.683) | 0.000 | 1.011 |
| Stroke | -0.240 | 0.082 | 8.569 | 0.787 (0.670 – 0.924) | 0.003 | 1.165 |
| Pressure ulcer, n (%) | 0.458 | 0.083 | 30.493 | 1.581 (1.344 – 1.860) | 0.000 | 1.046 |
| Psychogeriatric department | 0.248 | 0.122 | 4.118 | 1.281 (1.009 – 1.628) | 0.042 | 3.066 |
| Constant | -3.097 | 0.331 | 87.733 | - | - | - |

**Table 3.** Comparison of subjects with and without malnutrition admitted in 2013

|  | **Without malnutrition**  **(n=2,635)** | **With malnutrition (n=559)** | **Mean difference (95% CI)** | **Odds ratio**  **(95% CI)** |
| --- | --- | --- | --- | --- |
| Age, years (SD) | 81.5 (9.7) | 83.9 (8.6) | 2.4 (1.5 to 3.2)* | n.a. |
| Female, n (%) | 1,774 (67.3%) | 452 (80.9%) | n.a. | 2.1 (1.6 to 2.6)* |
| BMI, kg/m^2^ (SD) | 25.8 (4.4) | 18.6 (2.3) | -7.2 (-7.6 to 6.8)* | n.a. |
| Length of admission, days (median) | 701 (1060.9) | 687.0 (926.8) | p=0.129 (U-test) | n.a. |
| CDS Sum score | 39.2 (17.6) | 33.5 (17.2) | -5.7 (-7.3 to -4.1)* | n.a. |
| CDS item ‘eating and drinking’ | 2.7 (1.5) | 2.4 (1.4) | -0.3 (-0.5 to -0.2)* | n.a. |
| CDS item ‘communication’ | 3.3 (1.5) | 2.9 (1.6) | -0.4 (-0.5 to -0.3)* | n.a. |
| CDS item ‘mobility’ | 2.9 (1.6) | 2.5 (1.6) | -0.4 (-0.5 to -0.2)* | n.a. |
| Dementia, n (%) | 1,680 (63.8%) | 407 (72.8%) | n.a. | 1.5 (1.2 to 1.9)* |
| Diabetes mellitus, n (%) | 562 (21.3%) | 77 (13.8%) | n.a. | 0.6 (0.5 to 0.8)* |
| Diseases of the nervous system | 381 (14.5%) | 65 (11.6%) | n.a. | 0.8 (0.6 to 1.0) |
| Stroke | 655 (24.9%) | 116 (20.8%) | n.a. | 0.8 (0.6 to 1.0) |
| Respiratory diseases | 402 (15.3%) | 79 (14.1%) | n.a. | 0.9 (0.7 to 1.2) |
| Pressure ulcer, n (%) | 135 (5.2%) | 46 (8.3%) | n.a. | 1.7 (1.2 to 2.4)* |
| Type of department, n (%) |  |  |  |  |
| Psychogeriatric | 1,687 (64.0%) | 419 (75.0%) | n.a. | 1.7 (1.4 to 2.1)* |
| Somatic | 948 (36.0%) | 140 (25.0%) | n.a. | 0.6 (0.5 to 0.7)* |

**Table 4:** Multiple binary logistic regression analysis for factors associated with malnutrition of subjects admitted to nursing homes in 2013

| **Independent variables** | **Beta** | **Standard error** | **Wald statistics** | **OR (95% CI)** | **p-value** | **VIF** |
| --- | --- | --- | --- | --- | --- | --- |
| Age, years | 0.018 | 0.006 | 10.337 | 1.019 (1.007- 1.030) | 0.001 | 1.133 |
| Female | 0.543 | 0.120 | 20.543 | 1.720 (1.361 - 2.175) | 0.000 | 1.069 |
| CDS item ‘eating and drinking’ | -0.031 | 0.045 | 0.463 | 0.970 (0.888 – 1.059) | 0.496 | 1.752 |
| CDS item ‘communication’ | -0.070 | 0.041 | 2.960 | 0.932 (0.861 – 1.010) | 0.085 | 1.687 |
| CDS item ‘mobility’ | -0.112 | 0.038 | 8.496 | 0.894 (0.829 – 0.964) | 0.004 | 1.625 |
| Dementia | -0.133 | 0.189 | 0.495 | 0.876 (0.605 – 1.268) | 0.482 | 3.291 |
| Diabetes mellitus | -0.547 | 0.136 | 16.221 | 0.579 (0.443 – 0.755) | 0.000 | 1.012 |
| Stroke | -0.182 | 0.123 | 2.186 | 0.834 (0.656 – 1.061) | 0.139 | 1.123 |
| Pressure ulcer, n (%) | 0.490 | 0.187 | 6.852 | 1.632 (1.131 – 2.356) | 0.009 | 1.038 |
| Psychogeriatric department | 0.472 | 0.190 | 6.150 | 1.602 (1.104 – 2.326) | 0.013 | 3.192 |
| Constant | -3.564 | 0.497 | 51.490 | 0.028 | 0.000 |  |

**Table 5.** Comparison of subjects with and without malnutrition admitted in 2018

|  | **Without malnutrition**  **(n=2,450)** | **With malnutrition (n=478)** | **Mean difference (95% CI)** | **Odds ratio**  **(95% CI)** |
| --- | --- | --- | --- | --- |
| Age, years (SD) | 83.0 (9.6) | 84.3 (9.1) | 1.4 (0.5 to 2.3)* | n.a. |
| Female, n (%) | 1,677 (68.4%) | 369 (77.2%) | n.a. | 1.6 (1.2 to 2.0)* |
| BMI, kg/m^2^ (SD) | 26.4 (6.1) | 18.8 (2.6) | -7.6 (-7.9 to -7.3)* | n.a. |
| Length of admission, days (median) | 715 (1017) | 712 (1321) | p=0.921 (U-test) | n.a. |
| CDS Sum score | 43.5 (15.9) | 37.7 (16.5) | -5.7 (-7.3 to -4.1)* | n.a. |
| CDS item ‘eating and drinking’ | 3.2 (1.3) | 2.8 (1.4) | -0.5 (-0.6 to -0.3)* | n.a. |
| CDS item ‘communication’ | 3.6 (1.3) | 3.2 (1.4) | -0.4 (-0.6 to -0.3)* | n.a. |
| CDS item ‘mobility’ | 3.1 (1.5) | 2.7 (1.5) | -0.4 (-0.5 to -0.3)* | n.a. |
| Dementia, n (%) | 1,511 (61.7%) | 318 (66.5%) | n.a. | 1.2 (1.0 to 1.5) |
| Diabetes mellitus, n (%) | 515 (21.0%) | 75 (15.7%) | n.a. | 0.7 (0.5 to 0.9)* |
| Diseases of the nervous system | 229 (12.2%) | 64 (13.4%) | n.a. | 1.1 (0.8 to 1.5) |
| Stroke | 471 (19.2%) | 73 (15.3%) | n.a. | 0.8 (0.6 to 1.0) |
| Respiratory diseases | 372 (15.2%) | 81 (16.9%) | n.a. | 1.1 (0.9 to 1.5) |
| Pressure ulcer, n (%) | 206 (9.0%) | 62 (14.3%) | n.a. | 1.7 (1.2 to 2.3)* |
| Type of department, n (%) |  |  |  |  |
| Psychogeriatric | 1,542 (62.9%) | 335 (70.1%) | n.a. | 1.4 (1.1 to 1.7)* |
| Somatic | 908 (37.1%) | 143 (29.9%) | n.a. | 0.7 (0.6 to 0.9)* |

**Table 6:** Multiple binary logistic regression analysis for factors associated with malnutrition of subjects admitted to long-term care facilities in 2018

| **Independent variables** | **Beta** | **Standard error** | **Wald statistics** | **OR (95% CI)** | **p-value** | **VIF** |
| --- | --- | --- | --- | --- | --- | --- |
| Age, years | 0.014 | 0.006 | 5.456 | 1.014 (1.002 – 1.026) | 0.020 | 1.106 |
| Female | 0.374 | 0.128 | 8.552 | 1.454 (1.131 – 1.869) | 0.003 | 1.065 |
| CDS item ‘eating and drinking’ | -0.126 | 0.053 | 5.631 | 0.882 (0.794 – 0.978) | 0.018 | 1.723 |
| CDS item ‘communication’ | -0.142 | 0.048 | 8.832 | 0.867 (0.789 – 0.953) | 0.003 | 1.559 |
| CDS item ‘mobility’ | -0.067 | 0.045 | 2.235 | 0.935 (0.856 – 1.021) | 0.135 | 1.522 |
| Dementia | -0.279 | 0.204 | 1.870 | 0.757 (0.508 – 1.128) | 0.171 | 3.411 |
| Diabetes mellitus | -0.248 | 0.144 | 2.982 | 0.780 (0.589 – 1.034) | 0.084 | 1.009 |
| Stroke | -0.308 | 0.154 | 4.018 | 0.735 (0.544 – 0.993) | 0.045 | 1.083 |
| Pressure ulcer, n (%) | .0438 | 0.165 | 7.028 | 1.549 (1.121 – 2.141) | 0.008 | 1.062 |
| Psychogeriatric department | 0.353 | 0.208 | 2.883 | 1.424 (0.947 – 2.141) | 0.090 | 3.439 |
| Constant | -2.423 | 0.525 | 21.326 |  |  |  |
